# Emergency management of sport-related orofacial trauma

**DOI:** 10.1038/s41415-026-9542-9

**Published:** 2026-02-27

**Authors:** Geoffrey St George, Lyndon Meehan, Sally McCarthy

**Affiliations:** 859810901733828575698 Department of Endodontics, Royal National ENT and Eastman Dental Hospitals, 47–49 Huntley Street, London, WC1E 6DG, United Kingdom; 289098766298863001978 Swansea Bay University Health Board, Department of Restorative Dentistry, Port Talbot Resource Centre, Moor Road, Port Talbot, SA12 7BJ, United Kingdom; 430895224197491930785 Barts and The London School of Medicine, Queen Mary University London, Garrod Building, Turner Street, London, E1 2AD, United Kingdom

## Abstract

Sport-related orofacial trauma is a common occurrence, particularly in contact sports and among young adults, necessitating effective emergency management. This article highlights the prevalence of these injuries and emphasises the importance of proactive planning, including athlete and staff education, establishment of emergency protocols, and ensuring access to dental expertise. Immediate and appropriate on-site management, guided by the International Association of Dental Trauma guidelines, is crucial for optimal outcomes, especially in cases of avulsion. Furthermore, the complexities surrounding return-to-play decisions for elite athletes are discussed, advocating for a collaborative, individualised approach that prioritises long-term health and necessitates further research to establish specific guidelines. The evolving nature of dental trauma management in sports underscores the need for continuous professional development and a commitment to prioritising athletes‘ wellbeing.

## Key points

- Dental trauma has a high prevalence, highlighting the need for effective prevention and management strategies.
- It is important to be prepared and establish links with dental professionals to manage these often-stressful injuries effectively.
- Managing elite athletes requires individualised risk assessments and collaborative decisions with other members of the supporting team balancing the athlete's desire to return with the need to prioritise their long-term dental health, as specific return-to-play guidelines for dental trauma are lacking.

## Introduction

To many, sport dentistry is synonymous with dental trauma. Being a team dentist or sports dentist can be very rewarding and satisfying, especially when treating athletes who have sustained these injuries. It can foster collaboration with various dental specialists. This experience in collaborative trauma management provides a significant boost to technical proficiency and treatment planning, directly applicable to general dental practice. It also allows the development of professional relationships with sports team staff members and athletes. This can give a pleasant diversion from the hustle and bustle of day-to-day general practice. It also gives the dental healthcare professional the chance to be part of something special with their own local team or sports club.

## Prevalence of sport-related trauma

In a recent study,^1^ the estimated world-wide prevalence of dental trauma affecting the permanent dentition was 15.2% and the primary dentition was 22.7%. The incidence was estimated at 2.82 events per 100 persons per year. Therefore, over a billion people around the world may have had a traumatic dental injury.

For injuries caused by accidents, oral injuries represented 5% of all injuries sustained in individuals aged from birth till the age of 30 years.^2^ Interestingly, in the same study, it was found that those in the age group from 16–30 years sustained oral injuries most commonly at athletic sites.

There is great variation in the prevalence of dental trauma within each sport,^3^ depending on whether it is a contact sport (11.38%) or non-contact sport (5.24%).^4^ The prevalence in a number of popular sports can be seen in Table 1.^5^^,^^6^^,^^7^^,^^8^^,^^9^^,^^10^^,^^11^^,^^12^^,^^13^^,^^14^^,^^15^^,^^16^^,^^17^

**Table 1:** The prevalence of trauma in some popular sports

**Study**	**Sport**	**Assessment method**	**Type of trauma reported**	**Overall prevalence of dental trauma**
Ilia *et al.*^5^	Rugby	Supervised questionnaire	Orofacial	64.9%
Caglar *et al.*^6^	Ice hockey	Supervised questionnaire	Dental	29.72%
Ferrari *et al.*^7^	Jiu-jitsu Judo Skate hockey Basketball Handball Soccer	Interviewer-administered questionnaire	Dental	**28% overall** 41.2% 22.3% 11.5% 36.4% 37.1% 23.1%
Azodo *et al.*^8^	Basketball	Interviewer-administered questionnaire	Orofacial ‘mouth'	62.8% 6.4%
Dursun *et al.*^9^	Soccer	Self-reported	Orofacial	9.8%
Fasciglione *et al.*^10^	Inline skates	Interviewer-administered questionnaire	Dental	9.2%
Gass *et al.*^11^	Equestrianism	Interviewer-administered questionnaire	Dental	15.00%
Schmid *et al.*^12^	Mountaineering	Self-reported	Dental	1.33%
Persic *et al.*^13^	Squash	Interviewer-administered questionnaire	Orofacial ‘dental'	37.7% 4.5%
Gülses *et al.*^14^	Cycling	Data obtained from the ORBIS Information-Management-System for patients treated at a German department of oral and maxillofacial surgery	Dental	28.4%
Bolhuis *et al.*^15^	Hockey (field) – international athletes	Non-supervised questionnaire	Dental	20%
Pueringer *et al.*^16^	Tennis	National Electronic Injury Surveillance System (USA, using A&E hospital data from 100 departments)	Dental	1.5%
Ineichen *et al.*^17^	Alpine skiing	Non-supervised questionnaire based	Dental	23.6%

When looking at one sport in particular – boxing – prevalence varies across studies which is most likely due to differing assessment methods (Table 2).^18^^,^^19^^,^^20^^,^^21^

**Table 2:** The prevalence of dental trauma in boxing

**Study**	**Assessment method**	**Overall prevalence of dental trauma**
McCarthy *et al.*^18^	Supervised questionnaire, and clinical and radiographic examination (including cone beam computed tomography scan)	68.9%
Shirani *et al.*^19^	Clinical examination and radiographic examination (not including periapical views of teeth)	46.7%
Emerich *et al.*^20^	Non-supervised questionnaire based	35.9%
Ifkovits *et al.*^21^	Non-supervised questionnaire based	10.7%

Comprehensive examinations of athletes reveal higher dental trauma rates than questionnaires, suggesting underreporting in self-reporting studies.

## Injuries to teeth

Well-fitting mouthguards are a key element in the chain of prevention of dental trauma. Detailed guidance is outside of the scope of this article. However, recent recommendations have been published.^22^

**Fig. 1:**
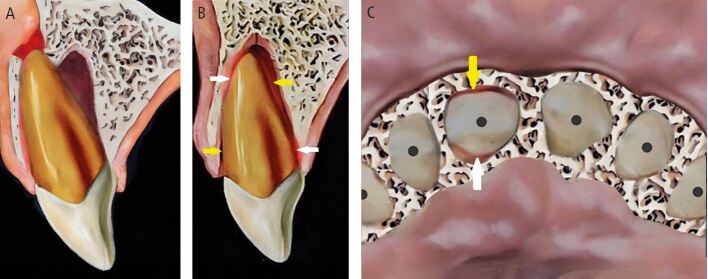
Dental trauma injuries resulting in (A) fracture of bone, and compression (white arrows) and tearing (yellow arrows) of the periodontal ligament in the sagittal (B) and axial (C) planes

**Fig. 2:**
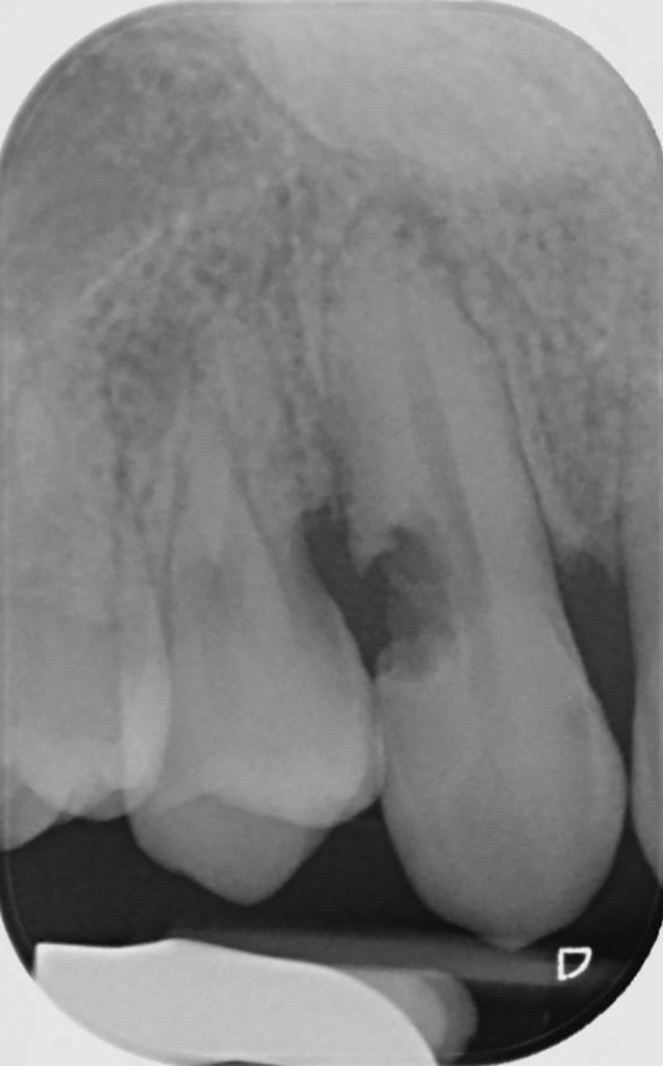
Resorption localised to a canine tooth following dental trauma

Dental trauma affects all tissues in the mouth, categorised by those involved: hard tissues/pulp, periodontal tissues, bone, and the gingiva/mucosa. Injury types include fractures, and compression and tearing of tissues (Fig. 1). Delayed or improper management also damages the pulp and periodontal ligament. Compressed tissues, like the periodontal ligament, often become necrotic and repair rather than regenerate, leading to resorption (Fig. 2).

## Planning ahead

While unexpected dental trauma is stressful, sports-related injuries can be managed through planning: education, readily available emergency care, and accessible follow-up care.

### Education

**Fig. 3:**
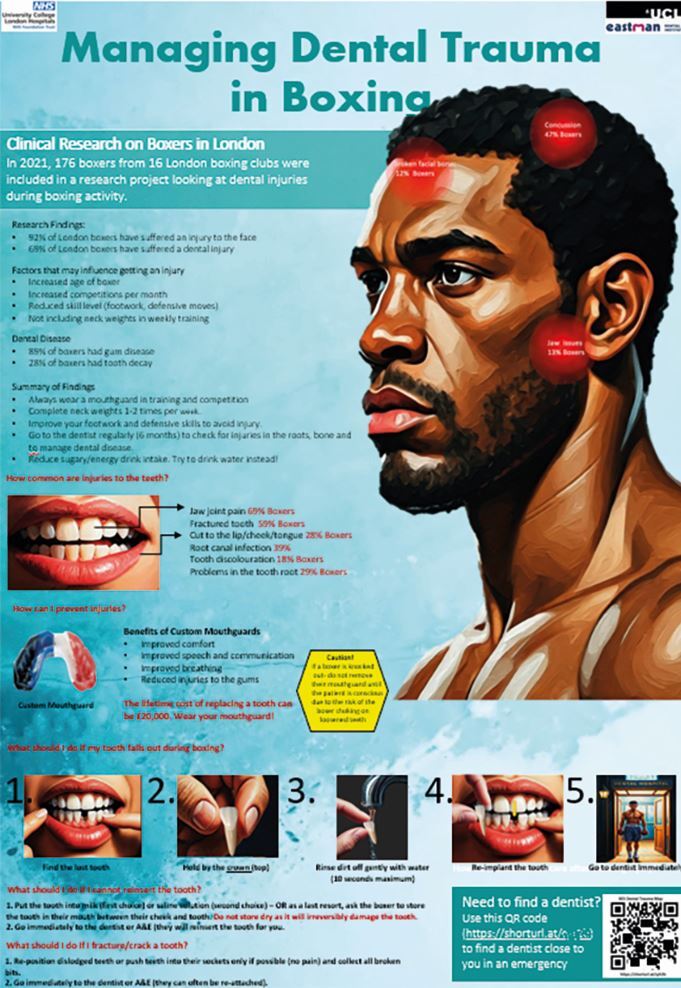
A boxing poster illustrating the results of a study^18^ used to educate boxers and their coaches on how to prevent and treat dental trauma and find a dentist (QR code) to treat them

An on-site dentist with experience in sports dental trauma management is ideal but uncommon due to logistical, financial, and access limitations. Therefore, educating medical and sporting colleagues on basic management is crucial, as knowledge is often lacking in dentists,^23^ doctors,^24^ athletes^25^ and coaches.^26^ However, studies show educational tools like pamphlets,^25^ apps,^25^ audio-visual aids^27^ and posters ^28^ (Fig. 3) can improve trauma care knowledge.

**Fig. 4:**
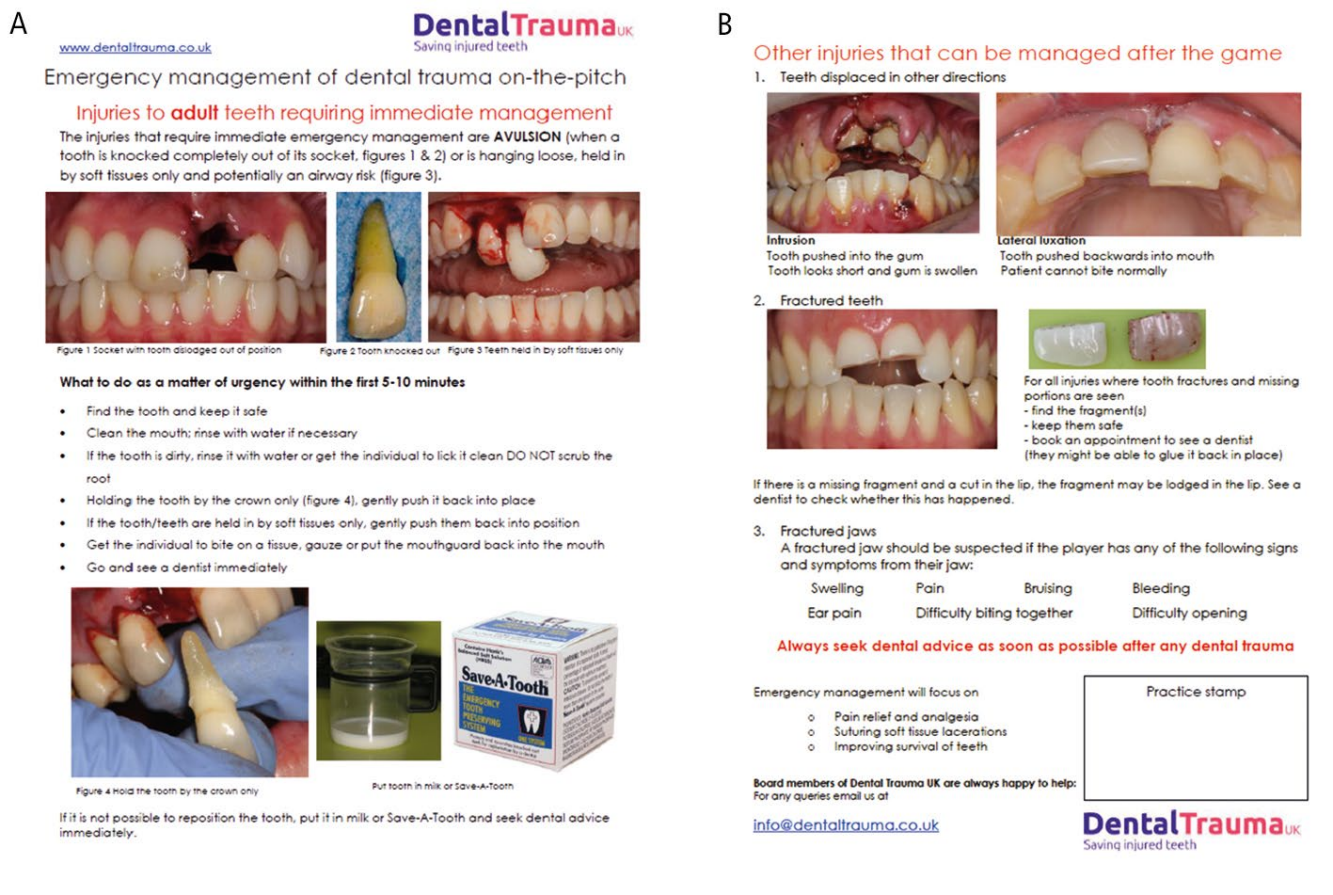
(A, B) The Dental Trauma UK pitch-side dental trauma flashcard (image used with permission from Dental Trauma UK)

New organisations like the UK Sports Dentistry Association and established societies such as the British Endodontic Society and Dental Trauma UK improve education and care. Dental Trauma UK's pitch-side flashcard (Fig. 4) provides vital immediate care guidance, which should be championed.

It is important to verify website and app accuracy for dental trauma advice. Inconsistencies do exist,^29^ though the International Association of Dental Trauma (IADT) endorsed apps are reliable.^29^ Sports' governing bodies must also ensure accurate guidance.

Artificial intelligence for dental trauma shows promise, but current accuracy is insufficient for clinical use^30^ requiring further research.

### Finding a dentist

Finding prompt dental care after trauma is challenging and stressful, especially away from home. For sports where injuries occur repeatedly, providing athletes with first aid and emergency care information prospectively greatly reduces stress.

Despite awareness of athlete oral health issues^31^^,^^32^ and national body recommendations,^33^ dental connections in sport could be better. The authors feel there is further scope to improve the standing of dentistry within sports medicine, by developing better links. Sporting groups should establish the following:

- A club dentist for comprehensive care (screening, education, trauma management and mouthguards)
- An emergency action plan for dental trauma assessment and treatment during training and competition.

Sports medicine staff should keep a contact list of dental professionals for immediate trauma advice.

**Fig. 5:**
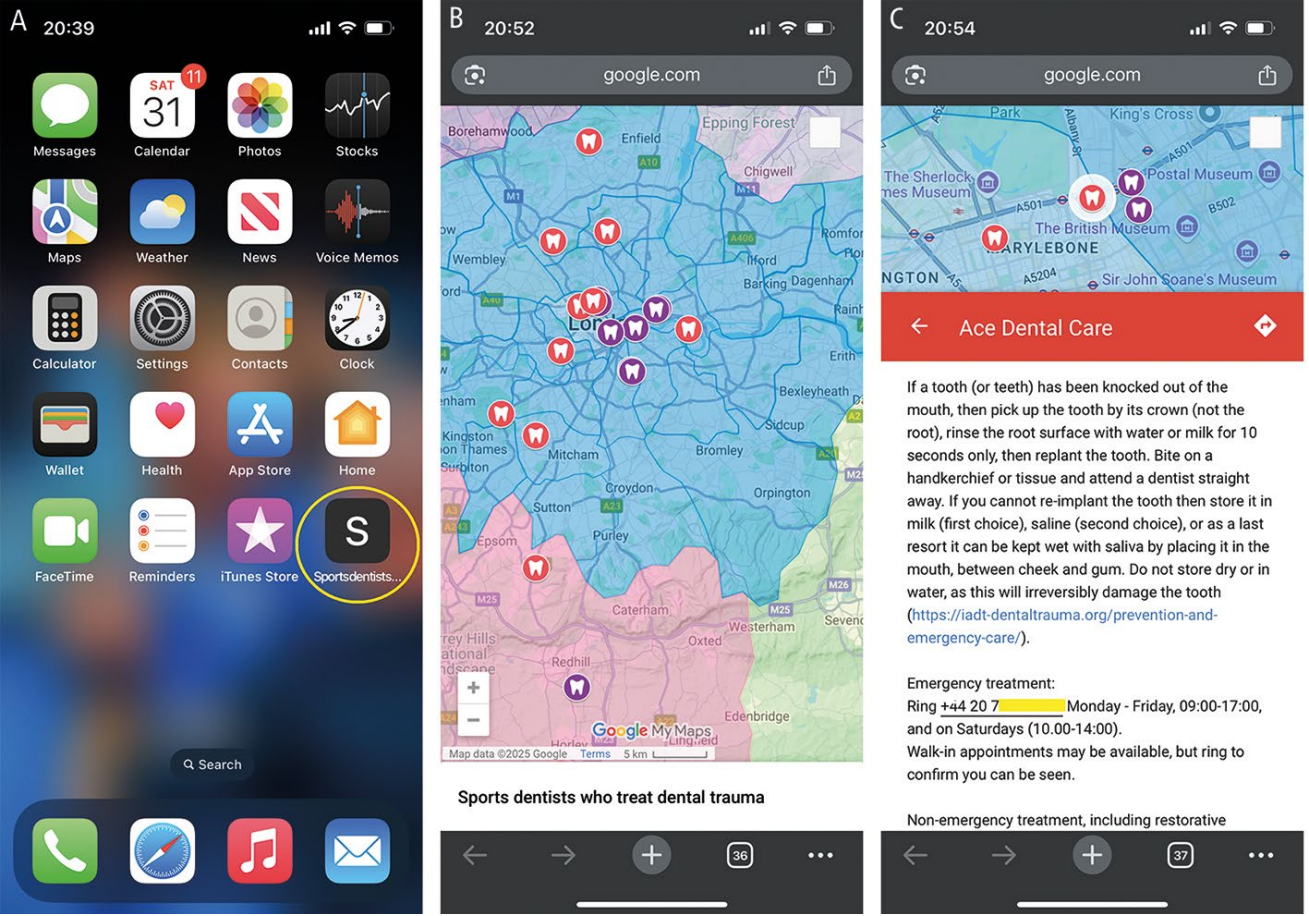
A custom dental trauma map saved as an icon on a mobile phone (A), can be used to find local dental clinics able to treat dental trauma (B), and contains all the details to make contact in an emergency (C)

It is relatively straightforward to create an online Google map displaying clinics that provide treatment for dental trauma. It can have fellow sports dentists' practices mapped onto it who can treat acute trauma and semi-acute trauma, and provide reconstructive treatment of injured athletes, with ongoing updates. The map can be saved as a mobile phone icon (Fig. 5).

**Fig. 6:**
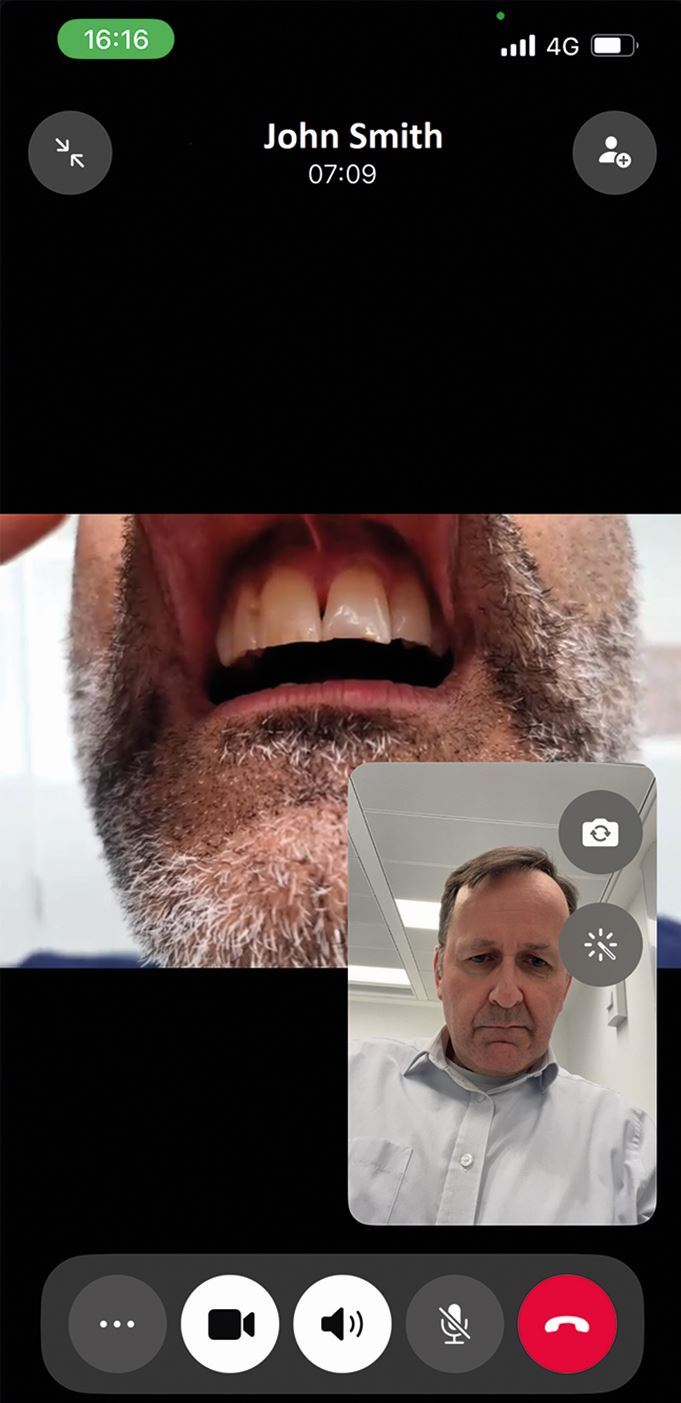
A WhatsApp video call used to assess trauma injuries remotely

Even when distant from an injured athlete, video calls can be used to aid assessment (Fig. 6).

Home team medical and dental staff must provide medical and dental support to visiting teams. This is important to remember when taking up a role with a club. Pre-match medical briefings may include dental scenario discussions.

## Emergency management of trauma

### Current guidelines

Dentists most often follow the IADT guidelines^34^^,^^35^^,^^36^^,^^37^ when managing dental trauma injuries. These have been produced since 2001 and then updated in 2007, 2012 and 2020, based on evidence and consensus. The 2020 revision includes notable changes which will be discussed later.

### Emergency management at the accident scene

Dental trauma, when it happens, may be managed by a dentist, team doctor, sports coach, other medical team member, or even another team member. Therefore, it is important that everyone is aware of what to do. Dentists should be aware of current guidelines. If there are facilities for treatment at the venue, then this can be performed. However, the treatment possible may be limited due to a lack of facilities, equipment and materials.

The initial assessment, called the primary survey, involves evaluating vital signs and addressing any life-threatening conditions. After stabilisation, a thorough examination, known as the secondary survey, is conducted including checks for facial and dental injuries.^38^

#### Assessment/treatment of general injuries

Patients should be screened for any serious injuries first, including head and neck, and other potentially life-threatening injuries. If suspected, medical staff should assess these. Otherwise, in the absence of medical staff, administer standard first aid^39^ and call the emergency services if required.

**Fig. 7:**
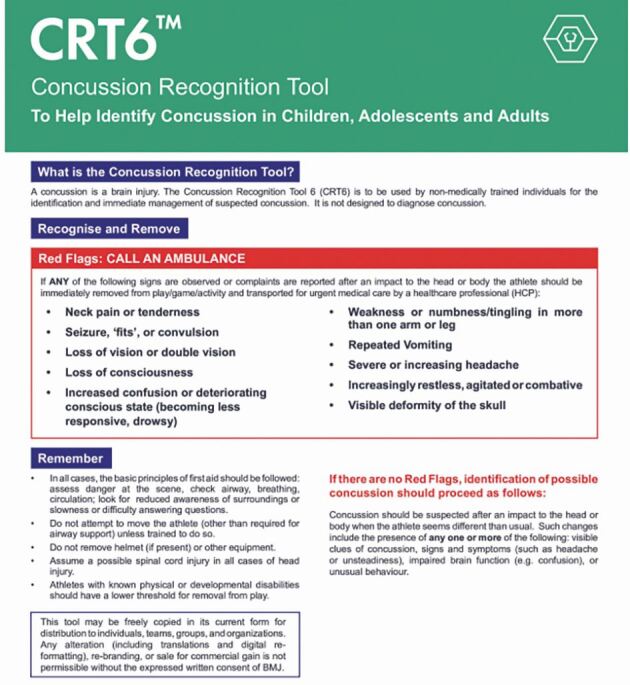
The CRT6, indicating red flags that should be recognised (image used with permission from Concussion in Sport Group [CISG])

**Fig. 8:**
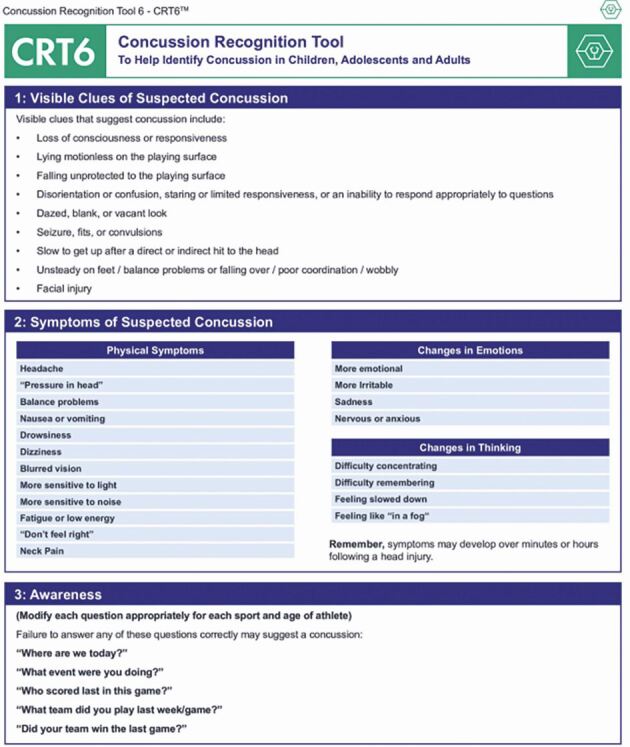
The CRT6, indicating signs and symptoms of concussion (image used with permission from Concussion in Sport Group [CISG])

Suspected head injuries can be assessed using one of the tools published in the *British Journal of Sports Medicine*. These include the sport concussion assessment tool (SCAT6)^40^ (adolescents/adults) or child SCAT6^4^^,^^41^ (8–12 years), though dentists may prefer the layperson-friendly concussion recognition tool 6 (CRT6).^42^ The CRT6's ‘red flags' (Fig. 7) necessitate immediate medical help, while its symptom checklist (Fig. 8) warrants removal from play and medical assessment.

#### Assessment/treatment of the mouth

**Fig. 9:**
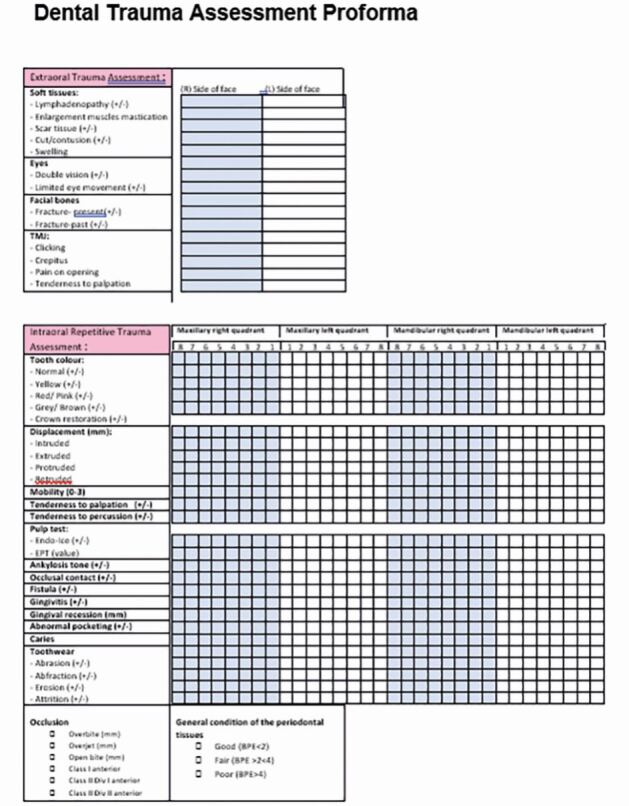
An example of a standardised dental trauma clinical assessment form

Examine the mouth's hard and soft tissues, occlusion, and dental health either on-site or in a medical room. Use a standardised form (Fig. 9) for documentation to avoid oversights. In rugby and Gaelic football, a 15-minute blood substitute allows for temporary player replacement, enabling on-field dental assessment and stabilisation.

#### Specific dental injuries

This article will only focus on the injuries needing urgent help and those where guidelines have change recently. Further details can be obtained from the current IADT guidelines.^34^^,^^35^^,^^36^^,^^37^

**Fig. 10:**
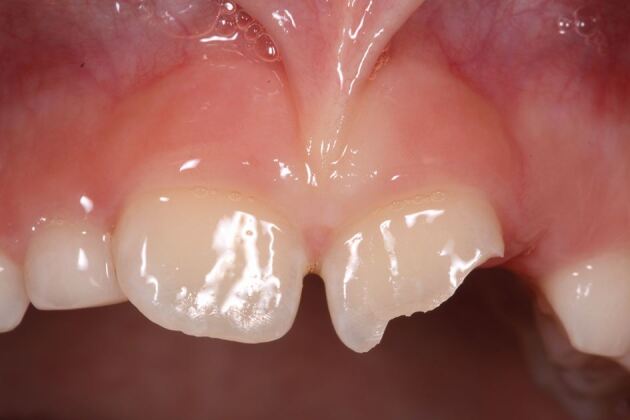
An uncomplicated enamel-dentine fracture

**Fig. 11:**
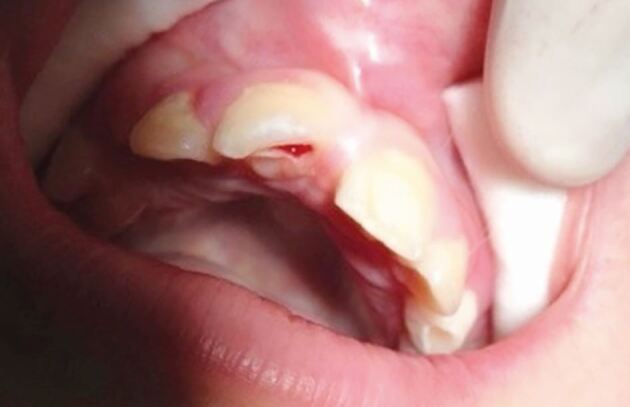
A complicated enamel-dentine fracture

**Fig. 12:**
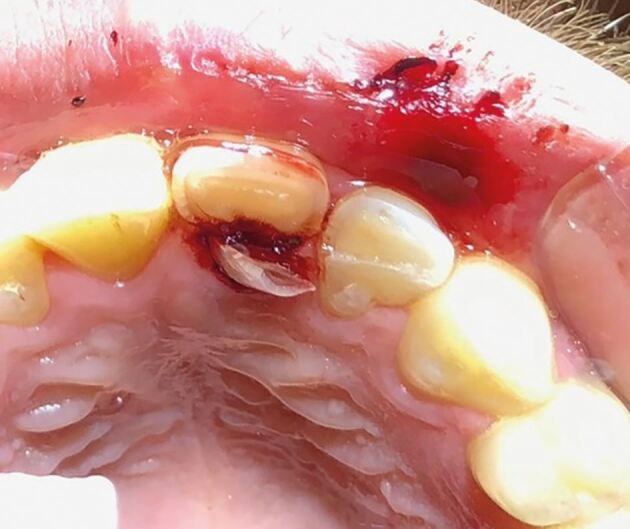
A complicated crown root fracture (21) and enamel-dentine fracture (22)

**Fig. 13:**
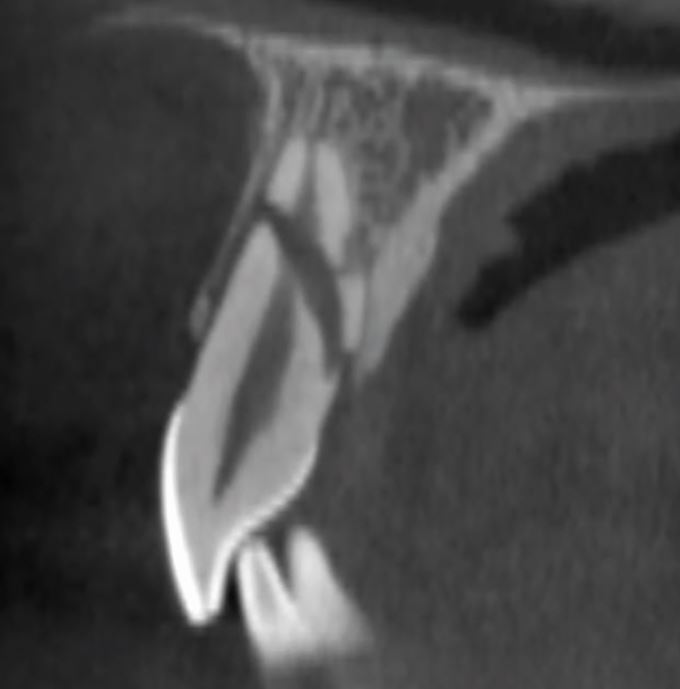
A CBCT scan of a root fracture, seen in the sagittal plane

**Fig. 14:**
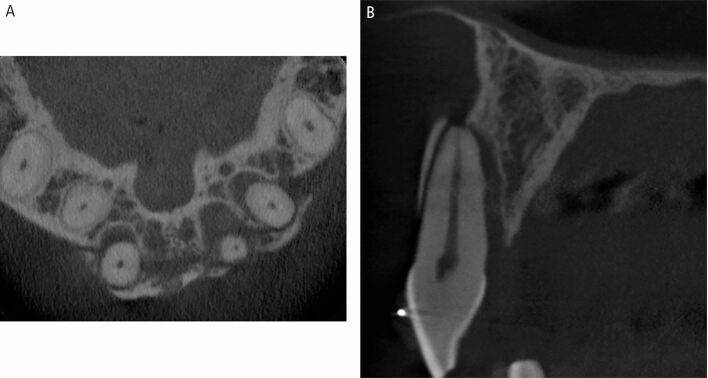
A CBCT scan of a dento-alveolar fracture extending over three maxillary incisor teeth, seen in the axial (A) and sagittal (B) planes

**Fig. 15:**
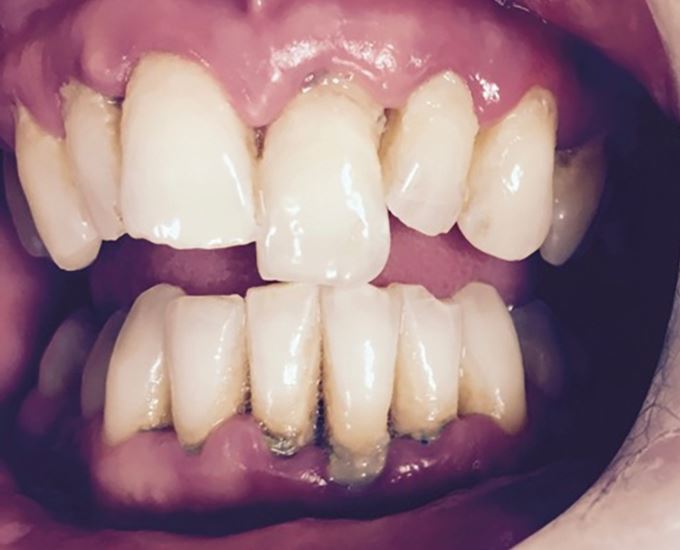
Extrusion of 21

**Fig. 16:**
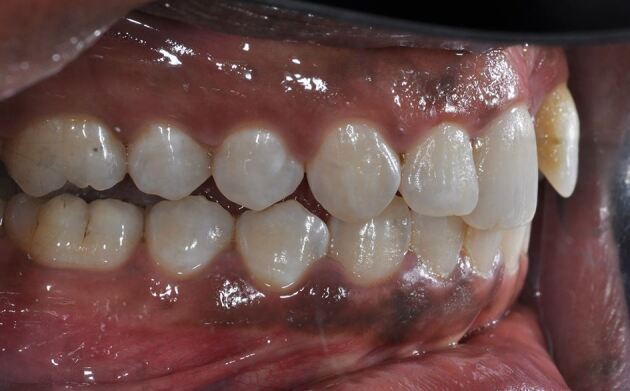
Lateral luxation of 11 and the resulting disclusion of the posterior teeth

**Fig. 17:**
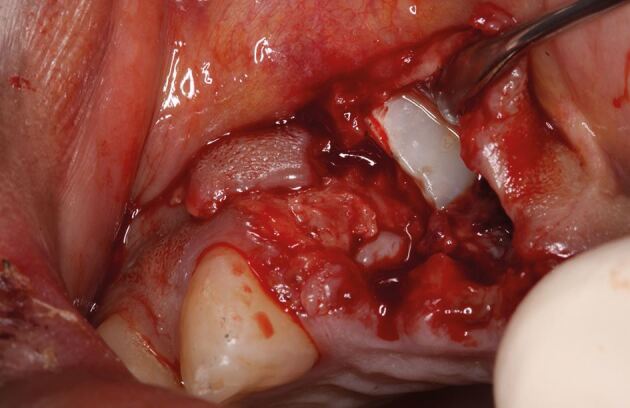
Intrusion of a central incisor, loss of a lateral incisor and laceration of the gingivae following trauma

Delays in treatment may occur from athlete behaviour (late arrival, underestimating urgency, uncooperativeness) and healthcare factors (staff knowledge, accident and emergency department waits, triage). Effective care follows three phases: acute (within three hours), subacute (24 hours), and delayed (over 24 hours), as categorised by Andreasen *et al.,*^43^ to be followed alongside IADT guidelines^34^^,^^35^^,^^36^^,^^37^ for structured management (Table 3; Figures 10, 11, 12, 13, 14, 15, 16 and 17).

**Table 3:** Dental trauma injuries, treatment, urgency and splinting times

**Injury**	**Management**	**Urgency**	**Splinting time**
Uncomplicated enamel-dentine fracture (Fig. 10) **Figure:** 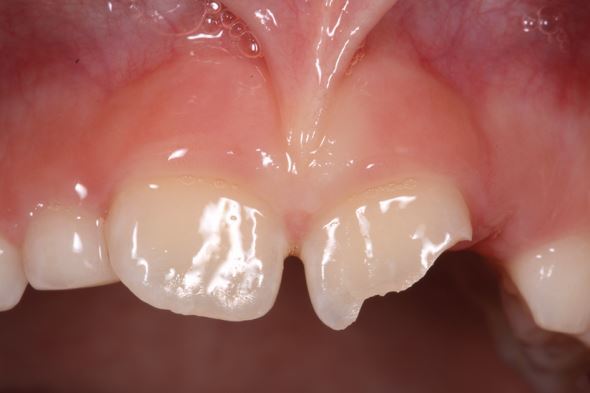	Recover tooth fragments and store in media for reattachment. If fragments are missing, restore the tooth with composite.	S D	Not applicable (NA)
Complicated enamel-dentine fracture (Fig. 11) **Figure:** 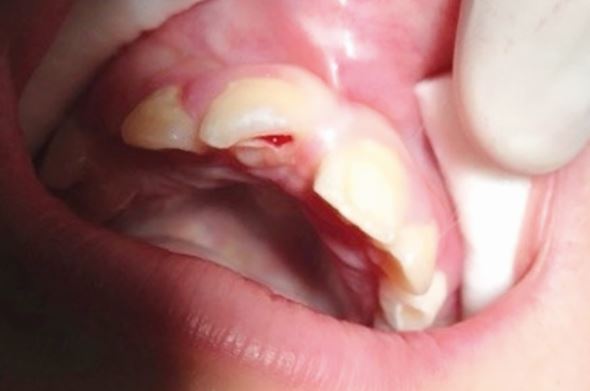	Pulp capping or partial pulpotomy.	S D	NA
Crown root fracture (Fig. 12) **Figure:** 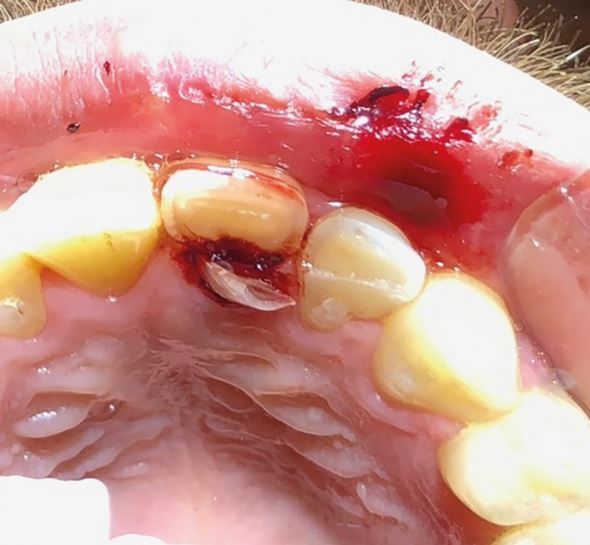	Coronal fragment removed, vital pulp treatment/pulpectomy and root canal treatment completed. May involve orthodontic/surgical extrusion to be restorable. Alternatively extract.	S D	NA
Root fracture (Fig. 13) **Figure:** 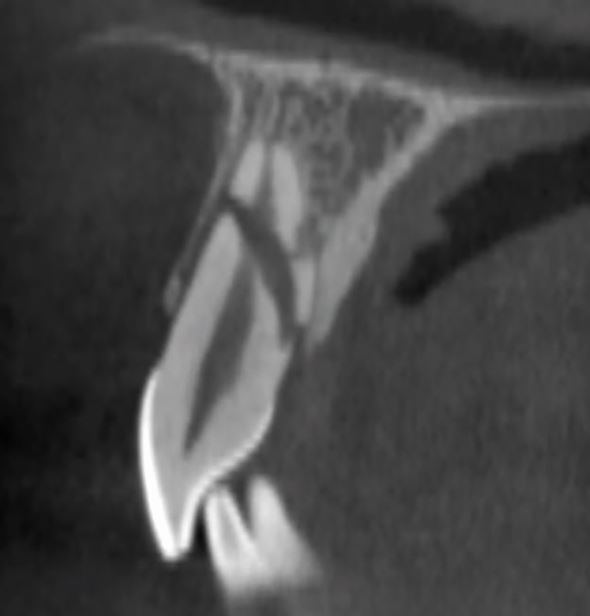	Reposition, if needed, and splint.	A S	Flexible splint for four weeks to four months (cervical)
Dento-alveolar fracture (Fig. 14) **Figure:** 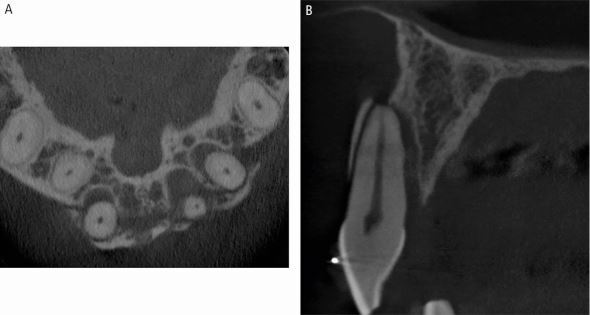	Reposition, if needed, and splint.	A	Flexible splint for two to four weeks
Concussion and subluxation	No treatment performed. Soft diet, pain relief.	NA	NA
Extrusion (Fig. 15) and lateral luxation (Fig. 16) **Figure:** 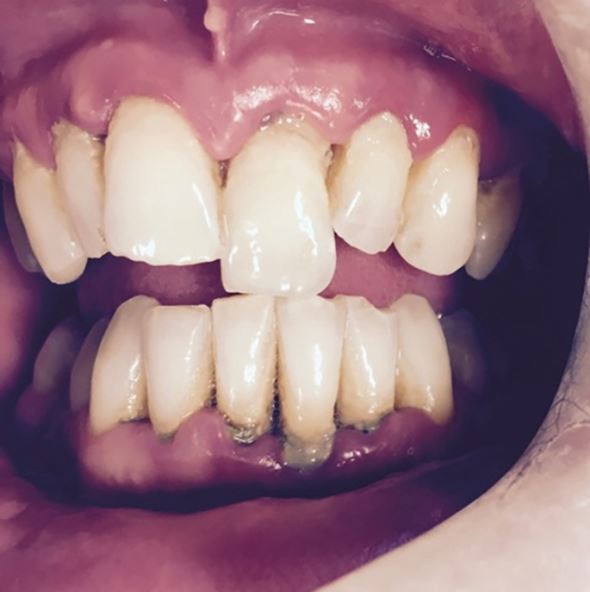 **Figure:** 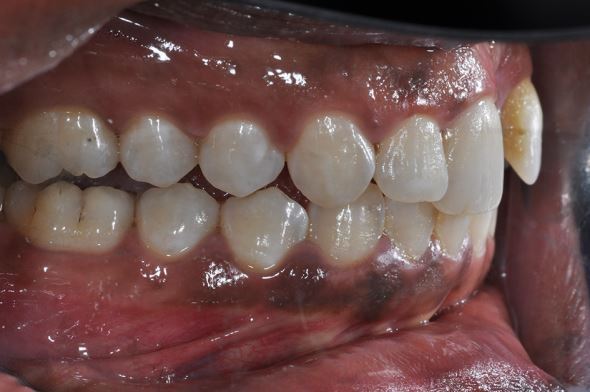	Reposition and splint.	A S	Flexible splint for four weeks
Intrusion (Fig. 17) **Figure:** 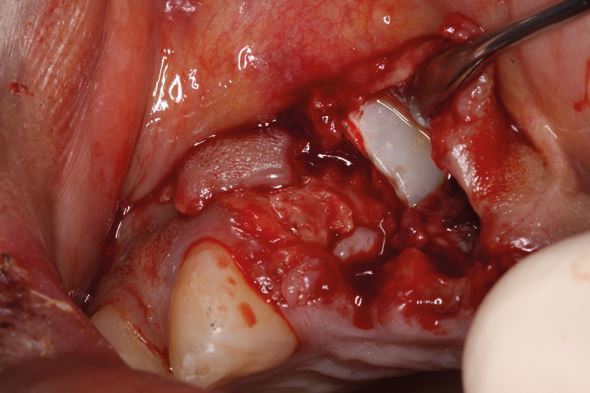	Monitor for re-eruption/ active repositioning.	S	Flexible splint for four weeks if surgically extruded.
Avulsion (Fig. 18) **Figure:** 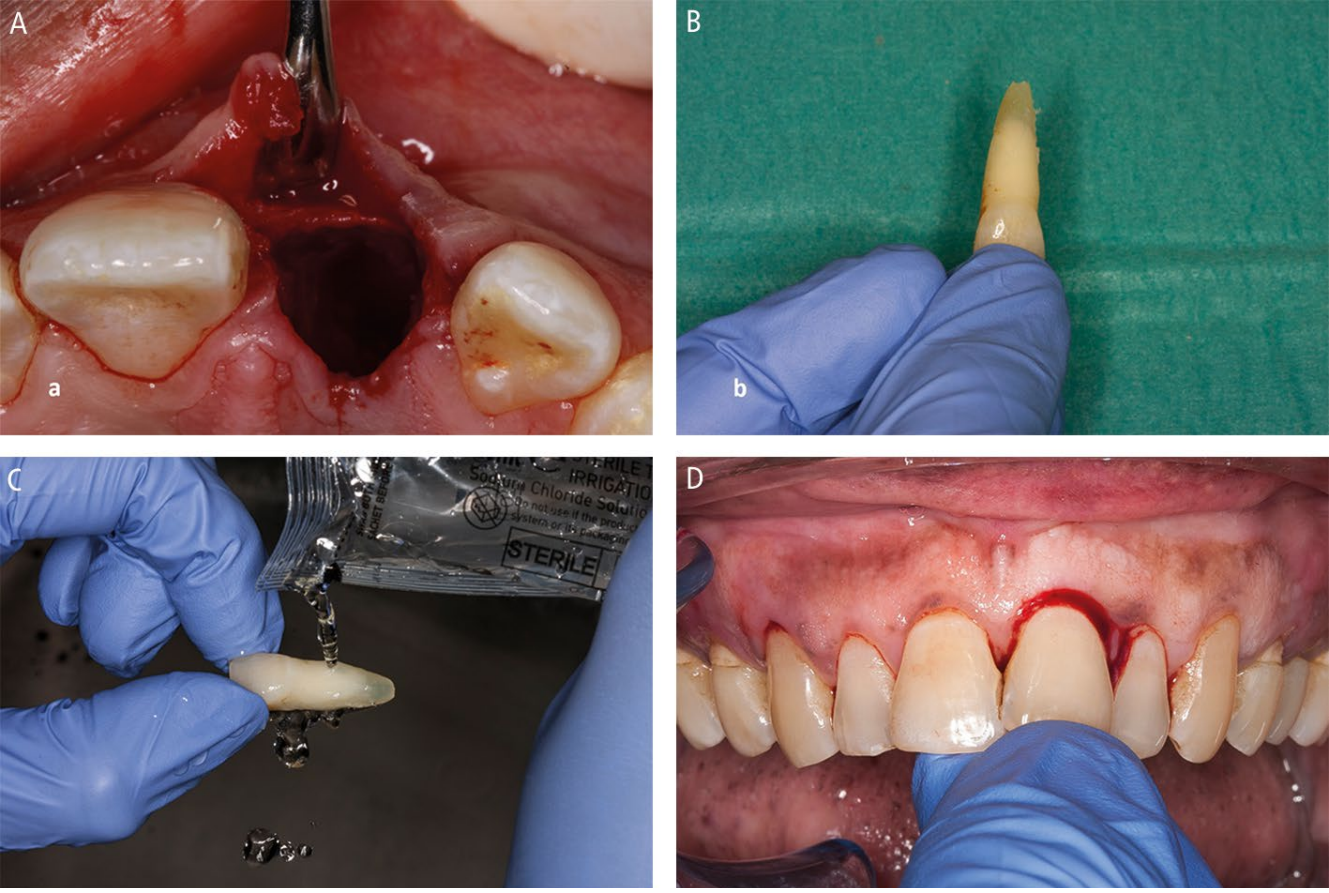	Replant and splint.	S	Flexible splint for two weeks
Injury to primary teeth	Subacute to delayed approach unless tooth causes occlusal problems (acute). Various treatments.	S D A	Flexible splint for two and four weeks for alveolar fractures and root fractures
Soft tissue injuries (Fig. 17)	Suture, monitor and keep clean depending on whether it is a laceration, contusion or abrasion.	A	NA
A, acute; D, delayed; NA, not applicable; S, subacute

### Replanting teeth

**Fig. 18:**
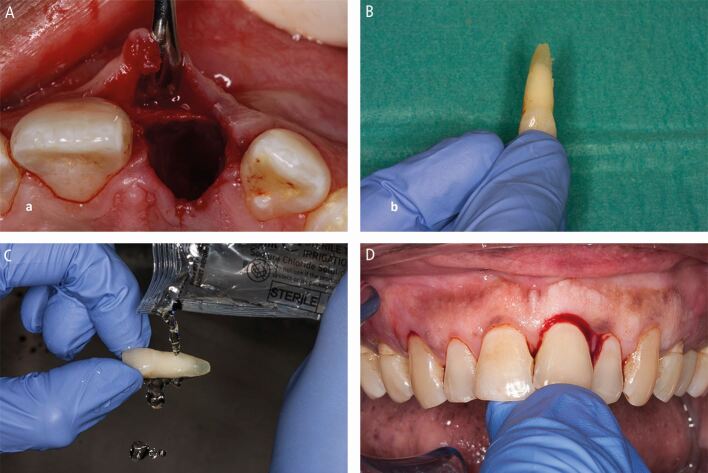
Replanting an avulsed tooth by (A) checking its socket contains no interferences to replantation, (B) picking it up by its crown, (C) rinsing it and (D) replanting it

Avulsion requires immediate attention as the periodontal ligament and pulp rapidly become necrotic outside of the protection of the tooth socket.^44^^,^^45^ That is if they aren't already necrotic due to the physical damage (compression and tearing) they sustain (Fig. 1) as they are avulsed. While other injuries may be severe, the fact that teeth remain in their sockets with these other injuries is a huge blessing. Immediate replantation is the treatment of choice,^36^ despite storage media options, due to the ‘wet time' (time in physiological media) impacting on prognosis.^46^ All versions of the guidelines have recommended the following (Fig. 18):

- Locate the avulsed tooth/teeth and pick it up by its crown
- Rinse the tooth briefly (ten seconds) to remove any debris
- Replant the tooth and bite on a handkerchief to maintain it in its position
- Visit the dentist immediately for treatment.

The 2020 guidelines^36^ recommend rinsing avulsed teeth with milk, saline, or saliva, not water. Saline is recommended as it is easily stored, while milk's availability varies due to it needing refrigeration, unless ultra-high temperature (UHT) processed milk is used. Saliva's viscosity makes it a poor rinsing agent, potentially delaying replantation.

The reason for excluding water, the most accessible liquid, is unclear. Despite the 2020 guidelines favouring milk, saline, or saliva, at the time of writing this article the IADT website (https://iadt-dentaltrauma.org/prevention-and-emergency-care/) and their SOS Tooth app, which can be viewed online (https://iadt-dentaltrauma.org/knocked-out/), recommend water. Supporting references from the guidelines recommend water ^47^ or relate to longer term storage media.^48^ Author correspondence also supported water. For a ten-second rinse, water remains a viable option if nothing better is available.

**Fig. 19:**
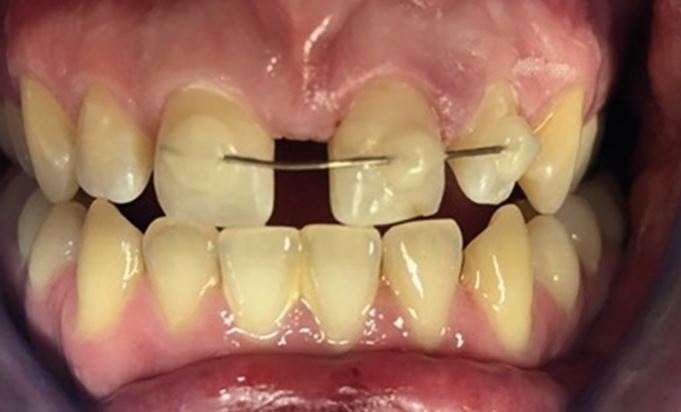
An orthodontic wire and composite dental trauma splint

After replantation, teeth need dental splinting and ideally in a surgery, but possibly on-site if facilities allow. Common splinting techniques are described from composite and orthodontic wire (<0.4 mm diameter) to novel approaches using fishing line or suturing across teeth.^49^ Splints should be easy to apply and remove, not interfere with occlusion, and allow access for endodontic treatment and oral hygiene measures (Fig. 19).

**Fig. 20:**
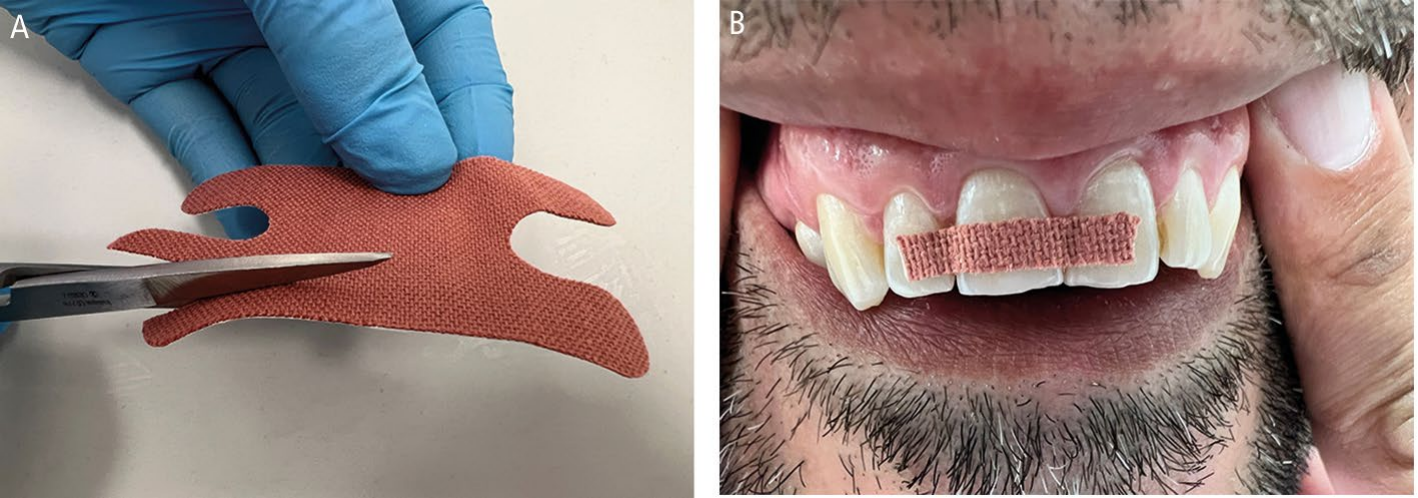
A Steroplast Premium waterproof plaster cut to size (A) and used as a temporary dental splint (B)

Ideal splinting may be difficult or impossible due to limited resources. Temporary options have included mouthguards, damp gauze shaped over the teeth, or clear orthodontic retainers until professional care is available. No commercially available splint exists, which can be attached to the teeth without the use of dental materials and equipment. However, as a last resort, a trimmed Steroplast Premium waterproof plaster (latex-containing) can temporarily secure avulsed teeth (Fig. 20) for several hours, but it is not a substitute for professional splinting.

**Fig. 21:**
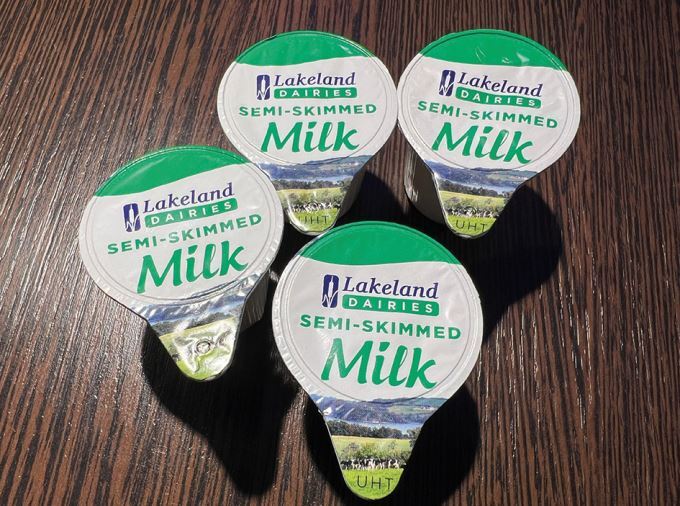
UHT milk can be used as a storage medium

If replantation is impossible (athlete is unconscious, concussed, uncooperative or when the socket wall is incompletely fractured), store the tooth in a suitable media.^50^ Commercial media (SaveA-Tooth, Dentosafe) are costly and difficult to obtain. Milk is readily available, but it needs refrigerating and allergy is possible,^51^ but rare. UHT milk, stored at room temperature, is a promising, affordable alternative (Fig. 21).^52^^,^^53^ Typically three 10–12 ml pots poured into a small container are normally sufficient to cover several teeth. If other media are unavailable, store the avulsed tooth in the athlete's saliva.

Two other IADT guideline changes in relation to avulsed teeth include tetracycline soaking^54^ for incompletely formed teeth (to increase the chances of pulp revascularisation and decrease the occurrence of resorption) and periodontal ligament removal^55^^,^^56^ for improperly stored or dry-stored teeth (to reduce the rate of replacement resorption). Both are no longer recommended due to lack of clinical benefit.

**Fig. 22:**
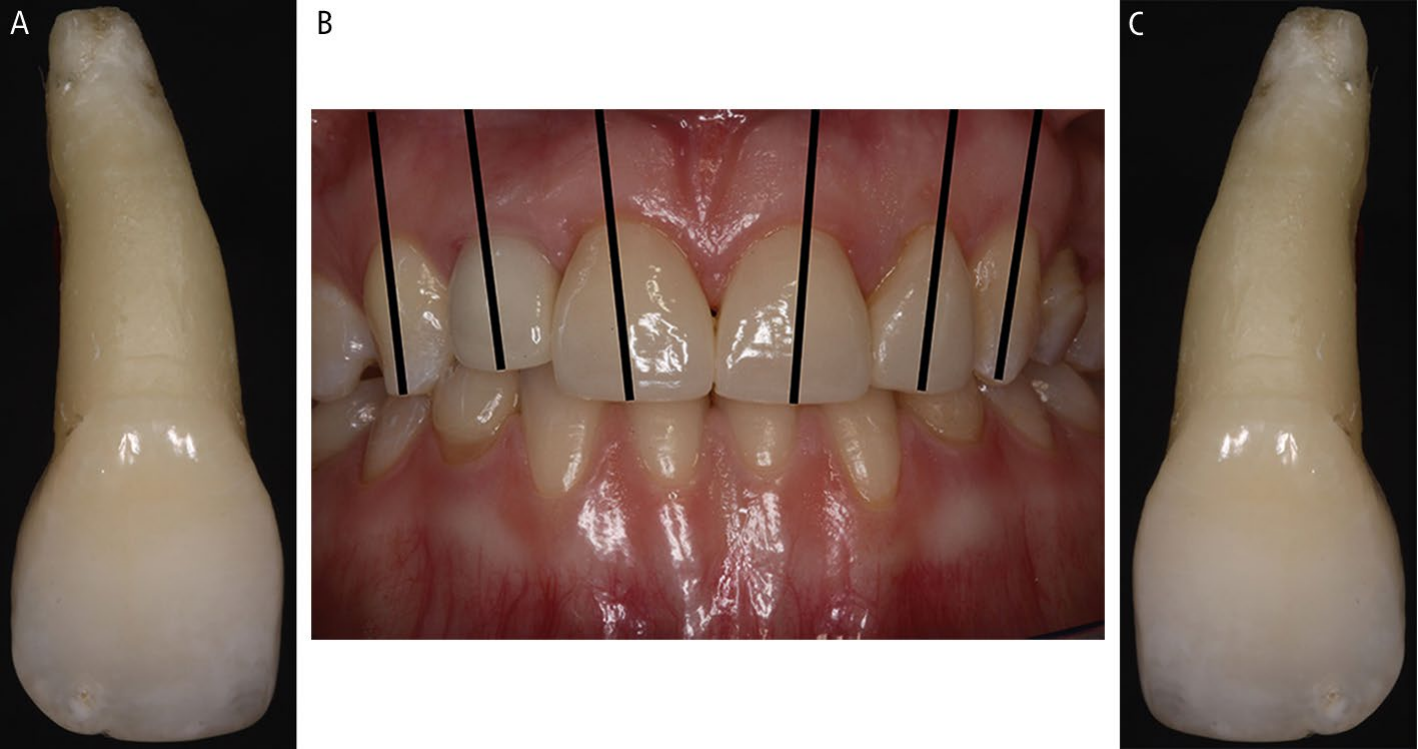
(A, B, C) To identify multiple avulsed teeth: maxillary centrals are largest, canines are ‘pointed', and root inclination distinguishes left from right
- Distinguishing deciduous from permanent teeth (treat as permanent if unsure and remove the tooth in the surgery if found to be a deciduous one)
- Managing loose teeth in mouthguards (leave in place until the mouthguard can be removed safely; translucent mouthguards facilitate this).

While dentists replant teeth easily, athletes and sports staff may face challenges when:

- Identifying multiple, avulsed teeth (Fig. 22)

Most sports dentists will have tales of treatment being prevented by coaches or athletes, especially in professional sports, where winning pressures prioritise immediate play over proper dental care. This can lead to delays in replanting avulsed teeth, risking tooth loss or complications from improper storage. While storage solutions exist, prompt replantation is crucial, and athletes and medical staff must understand the risks of delaying treatment.

### Collecting fractured teeth

Any teeth fragments should be accounted for, to confirm they are not embedded in lips, swallowed or inhaled. These should be stored wet and can be reattached to broken teeth in the dental surgery.

### Complicated crown fractures

Partial pulpotomy and restoration of the tooth is the treatment of choice when a dental pulp is exposed. The classic study by Cvek^57^ has often been interpreted as time between pulp exposure and treatment, or the size of the pulp exposure not impacting on the success of the partial pulpotomy. However, optimal success depends on treating teeth quickly (less than nine days), and when the exposure is less than 4 mm².^58^ It is also more successful in teeth with open apices.^58^ As long as the treatment is performed following these guidelines, then immediate treatment at the athletic site is not required.

All the above treatment assumes that the injured athlete will receive further, definitive care from their dentist shortly after the emergency treatment.

A few other injuries have undergone revision of their guidelines by the IADT and the evidence for and against these have been appraised in other journal articles.^59^^,^^60^

### Complicated crown-root fractures

While 2012 guidelines^61^ favoured partial pulpotomy for exposed pulps, 2020 guidelines^35^ suggest pulpectomy followed by root canal treatment. This contrasts with growing evidence supporting vital pulp therapy, even in teeth with caries, and partial pulpotomy success in crown fractures.^57^

### Intrusion injuries of immature teeth

Guidelines for intruded immature teeth have changed. The 2012 guidelines^61^ recommended monitoring minor intrusions (<7 mm) and active repositioning for severe cases (>7 mm). The 2020 guidelines^35^ suggest monitoring all intrusions, regardless of severity. A recent review^59^ advocates case-by-case management based on intrusion severity, reflecting a lack of consensus in the literature regarding monitoring^62^ versus active repositioning.^63^

### Lateral luxation

The 2020 IADT guidelines^35^ recommend immediate root canal treatment in teeth with complete root formation that have been laterally luxated, assuming pulp necrosis will occur (‘the pulp will likely become necrotic' and that ‘root canal treatment should be started'). This contrasts with the 2012 guidelines^61^ that state ‘if the pulp becomes necrotic, root canal treatment is indicated to prevent root resorption'. However, a recent systematic review ^60^ showed pulp necrosis can be less than 60% in teeth with mature apices, and suggested diagnosis and then treatment should be based on discoloration, negative pulp testing, and radiographic changes, rather than pre-emptive treatment.^64^

## Medical room and match day dental kit

**Fig. 23:**
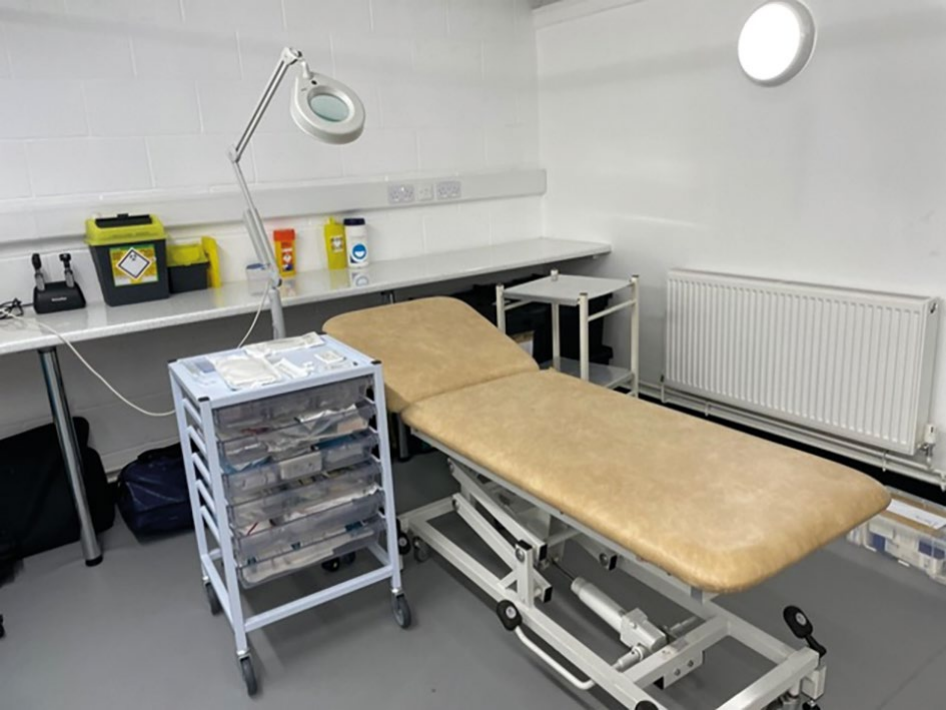
A typical sports medical room, where patients can be examined

**Fig. 24:**
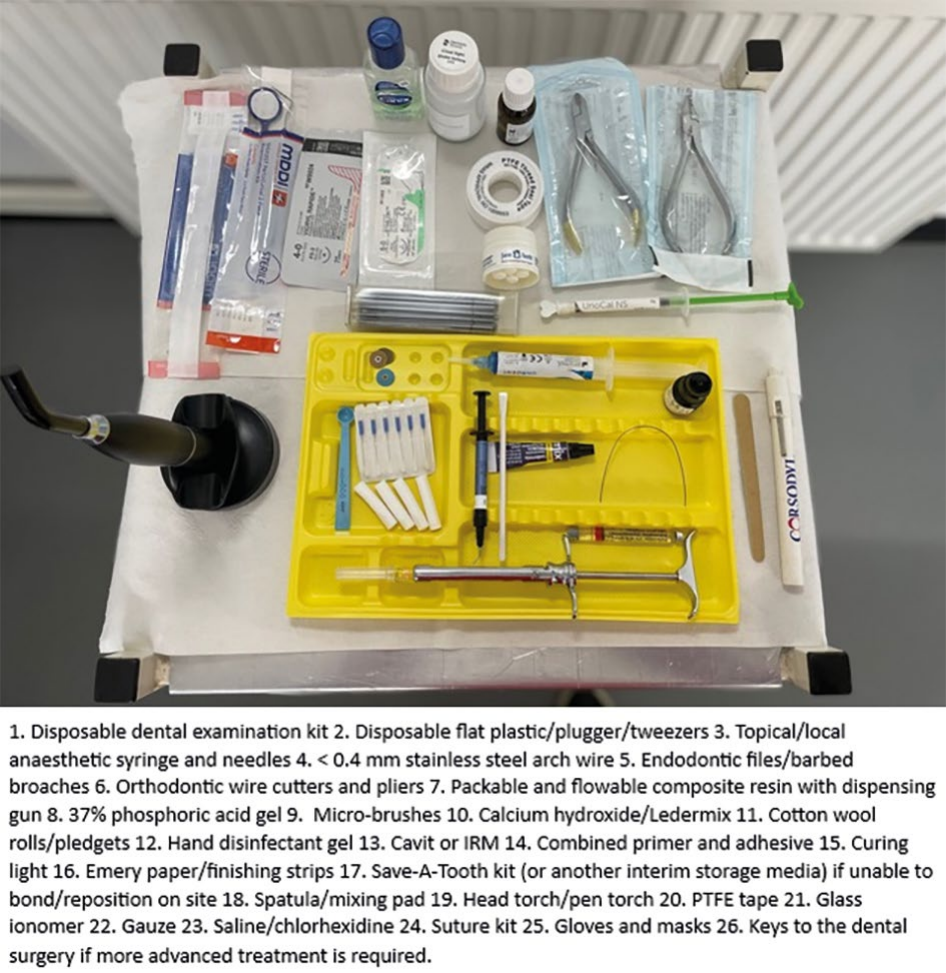
Typical dental equipment and materials that need to be available on a competition day

Elite sports require a private standardised medical room (Fig. 23) with hygienic space, proper lighting, a clean examination table, and essential supplies like gloves and sharps disposal.^65^ On match days, dental personnel must assess their ability to treat injuries in unfamiliar settings, considering environment, time, and equipment. If inadequate, they should stabilise and refer the athlete. A matchday dental first aid kit is essential and Figure 24 shows some suggested materials and instruments; but this is not an exhaustive armamentarium.

### Return to play considerations

For elite athletes, sport is both a passion and an income, tied directly to performance. They face packed schedules and may rush recovery post-injury, fearing lost performance or position. A crucial question they may ask is ‘how soon can I play again?' While athletes may seek a quick return to play, dental professionals should prioritise their wellbeing, resisting external pressures.

No return-to-play guidelines exist for dental trauma in elite sports. Spinas *et al.*^66^ found timely returns didn't increase long-term complications, varying by sport and injury. Sports dentistry associations should create consensus guidelines. Anecdotally, many elite rugby players return within a week of luxation, root, or alveolar fractures, following repositioning, splinting, and use of a triple laminate mouthguard.

We should consider the consensus papers by Fardy, Fowell, Hayton and Scott *et al.* on return-to-play timeframes for elite athletes with maxillofacial injuries, recognising that their management differs from that of the general public.^67^^,^^68^^,^^69^^,^^70^

Individualised player risk assessments are crucial before return to play, ensuring safety and reducing injury risk. Key questions include sport-specific risks, athlete's role and demands, treatment type, recovery plan, impact of pain/swelling, championship context, and concurrent injury management.

Collaborative discussions with the athlete and sports medicine team regarding early return risks are crucial. The dentist shares responsibility and must prioritise athlete health. Discuss adjunctive roles with indemnity providers. Adherence to General Dental Council standards is mandatory.

## Conclusion

The management of dental trauma in athletes can be very rewarding, but there are a number of challenges. However, many of these can be overcome by forward planning and ensuring you keep up to date with this continually evolving area of dentistry. When challenges can't be overcome, then athlete and dentist health should be prioritised to ensure successful outcomes.
